# Staphylococcus aureus N-terminus formylated δ-toxin tends to form amyloid fibrils, while the deformylated δ-toxin tends to form functional oligomer complexes

**DOI:** 10.1080/21505594.2021.1928395

**Published:** 2021-05-24

**Authors:** Xinyu Zhou, Yuling Zheng, Qingyu Lv, Decong Kong, Bin Ji, Xuelian Han, Dongsheng Zhou, Zeyu Sun, Li Zhu, Peng Liu, Hua Jiang, Yongqiang Jiang

**Affiliations:** a State Key Laboratory of Pathogens and Biosecurity, Institute of Microbiology and Epidemiology, Beijing, China; b Institute of Nano Biomedicine and Engineering, Department of Instrument Science and Engineering, School of Electronic Information and Electrical Engineering, Shanghai Jiao Tong University, Shanghai, China; c Beijing Institute of Biotechnology, Beijing, China

**Keywords:** *Staphylococcus aureus*, CA-MRSA, δ-toxin, PSMα, amyloid fibrils, oligomer, complex, formylation, deformylation

## Abstract

The community-associated Methicillin-resistant *Staphylococcus aureus* strain (CA-MRSA) is highly virulent and has become a major focus of public health professionals. Phenol-soluble modulins (PSM) are key factors in its increased virulence. δ-Toxin belongs to PSM family and has copious secretion in many *S. aureus* strains. In addition, δ-toxin exists in the *S. aureus* culture supernatant as both N-terminus formylated δ-toxin (fδ-toxin) and deformylated δ-toxin (dfδ-toxin) groups. Although δ-toxin has been studied for more than 70 years, its functions remain unclear. We isolated and purified PSMs from the supernatant of *S. aureus* MW2, and found fibrils and oligomers aggregates by Size Exclusion Chromatography. After analyzing PSM aggregates and using peptide simulations, we found that the difference in the monomer structure of fδ-toxin and dfδ-toxin might ultimately lead to differences in the aggregation ability: fδ-toxin and dfδ-toxin tend to form fibrils and oligomers respectively. Of note, we found that fδ-toxin fibrils enhanced the stability of biofilms, while dfδ-toxin oligomers promoted their dispersal. Additionally, oligomeric dfδ-toxin combined with PSMα to form a complex with enhanced functionality. Due to the different aggregation capabilities and functions of fδ-toxin and dfδ-toxin, we speculate that they may be involved in the regulation of physiological activities of *S. aureus*. Moreover, the dfδ-toxin oligomer not only provides a new form of complex in the study of PSMα, but also has significance as a reference in oligomer research pertaining to some human amyloid diseases.

## Introduction

As a major human pathogen, *Staphylococcus aureus* causes numerous infectious diseases, including skin and soft tissue infections (SSTI), bacteremia, sepsis, pneumonia, and osteomyelitis [1,2]. *S. aureus* has had the remarkable ability to acquire resistance to any antibiotic yet devised [3], and the development of a universal *S. aureus* vaccine has so far proceeded poorly. Thus, treatment of *S. aureus* infections has become more difficult. In contrast to hospital-associated Methicillin-resistant *S. aureus* (HA-MRSA) infections, for which there is a predisposing risk factor or condition, community-associated (CA-MRSA) infections can occur in otherwise healthy individuals [4,5]. Importantly, the observation of a number of CA-MRSA strains having emerged internationally suggests that CA-MRSA strains are more virulent and transmissible than HA-MRSA strains [6]. Therefore, CA-MRSA has become a major focus of infection control efforts globally [7].

δ-toxin, which was originally reported in 1947 [8] – also known as δ-hemolysin and δ-lysin – is a widely-known peptide produced by various *Staphylococcus* strains. It is an α-helical amphipathic 26 amino acid peptide. It is encoded within RNAIII, the regulatory molecule of the accessory gene regulator (Agr) quorum-sensing system [9]. δ-toxin belongs to the peptide toxin family of phenol-soluble modulins (PSMs). Besides δ-toxin (PSMγ), PSM also includes the shorter (~20 amino acids) PSMα1-4, the longer (~40 amino acids) PSMβ1-2 which encoded on the genome, and the PSM-mec which encoded on the Mobile genetic element (MGE) [10]. Wang *et al*. pointed out that increased secretion of PSMs may be one of the reasons for the increased virulence of CA-MRSA [11]. PSMα has the most strongly cytolytic activity among PSMs, and can provoke the expression of proinflammatory cytokines, kill competing microbes, recruit, activate, and destroy neutrophils after phagocytosis, and facilitate the structuring and detachment of biofilms [12,13]. Thus, it is regarded as the vital virulence factor in the PSM family.

In contrast to PSMα, δ-toxin has been known for many years, but its specific functions in *Staphylococcus* physiology and pathogenesis have remained largely obscure. δ-toxin lyses erythrocytes as well as other mammalian cells [14], and displays limited antimicrobial activity against bacteria [15]. A special function discovered later is that the δ-toxin triggers mast cell degranulation and causes significant contribution to the development of the atopic dermatitis [16]. The δ-toxin is usually the most strongly produced PSM peptide, and in many strains is by far the most abundant secreted protein [11,17]. Therefore, we suspect that it has other important unknown functions. Regardless, δ-toxin of *S. aureus* translation initiates with formyl-methionine, and the N-terminal formyl can be removed by peptide deformylase (PDF, encoded by *pdf1* in *S. aureus*). Thus, δ-toxin accumulates in culture medium in formylated (fδ-toxin) and deformylated (dfδ-toxin) forms [18]. However, the precise functions of the two δ-toxins in *S. aureus* pathogenesis have yet to be elucidated. Some studies reported they have no differences in lysis and mast cell degranulation activity, just some diversity in attracting and inducing the release of IL-8 toward neutrophils [14,18,19].

In our previous study on the PSMs of CA-MRSA, we found that synthetic PSMα4 has highly hydrophobic [20,21], while a certain amount of soluble PSMα4 was present in the supernatant of the CA-MRSA strains [11]. In addition, when we tried to obtain PSMs from the supernatant with the ultrafiltration tube (~50 kDa), we could not get any PSMs in the filtrate (data not shown). Therefore, we assumed that to some extent, PSMs might exist in the form of aggregates in the supernatant of *S. aureus*. In this study, we isolated and purified PSMs from the supernatant of *S. aureus* MW2, and separated fibrils and oligomers aggregates by SEC. After analyzing PSM aggregates and using peptide simulations, we found the aggregation of δ-toxin: fδ-toxin tends to form fibrils, while dfδ-toxin tends to form oligomers. Of note, dfδ-toxin oligomer will form a complex with PSMα, and when the dfδ-toxin oligomer forms a complex with PSMα, the cytolytic activity of PSMα is significantly enhanced. This results propose a new pattern for how PSMs function. This would provide a new understanding of the virulence of CA-MRSA.

## Materials and methods

### Peptides and reagents

Peptide sequences:

PSMα1:fMGIIAGIIKVIKSLIEQFTGK

PSMα2:fMGIIAGIIKFIKGLIEKFTGK

PSMα3:fMEFVAKLFKFFKDLLGKFLGNN

PSMα4:fMAIVGTIIKIIKAIIDIFAK

fδ-toxin: fMAQDIISTISDLVKWIIDTVNKFTKK

dfδ-toxin: MAQDIISTISDLVKWIIDTVNKFTKK

The peptides were synthesized according to the previous report [11] by Sangon Biotech. The purity of the peptides was > 95%. Trifluoroacetic acid (TFA), hexafluoroisopropanol (HFIP), and Thioflavin T (ThT) were purchased from Sigma-Aldrich. Bis(sulfosuccinimidyl) suberate (BS3), and dithiothreitol (DTT) were purchased from Thermo Fisher Scientific. Ultra-pure water was purchased from Millipore Sigma.

### Peptide pretreatment

Peptide pretreatment was determined as described with slight modification [22]. Lyophilized powder of peptides was freshly dissolved in TFA-HFIP (1:1), to a concentration of 1 mg/mL, sonicated for 10 min in a sonication bath, and then evaporated by using a centrifugal vacuum concentrator (miVac) for 1 day. Unless immediately tested, the treated peptides were stored at −20^o^C.

### Bacterial strains and grow condition

The MW2 strain (USA400, ST1 lineage), typically associated with community outbreaks [23], which can cause severe sepsis in humans [24], is representative of the USA400 group of organisms. *S. aureus* MW2 and its ΔPSMα, Δδ-toxin strains [11,25] were gifts from Dr. Min Li (Shanghai Jiao Tong University). Bacteria were grown in tryptic soy broth (Oxoid).

### Purification of PSMs from MW2 supernatant

Purification was performed using the previously described method [21] or a new method established in this study. MW2 and mutants were aerobically cultured for 24 h in tryptic soy broth (TSB) at 37°C. The bacterial culture was centrifuged at 8000 × g for 10 min and the supernatant was collected, and filtered through a 0.22 μm membrane to completely remove bacteria. Ammonium sulfate was added to the supernatant to achieve 75% saturation. The mixture was incubated at 25°C for 6 h, then the precipitate was collected after centrifugation at 8000 × g for 10 min, dissolved in PBS, then mixed with 100% ethanol to reach a final ethanol concentration of 80% (v/v), incubated at 25°C for 10 min, and the mixture was centrifuged at 8000 × g for 10 min. The ethanol-soluble fraction was dried using a vacuum centrifugal concentrator (miVac) at 25°C for 12 h. Unless immediately tested, the products were stored at −20°C.

### Size exclusion chromatography (SEC)

Protein sample was injected into a Superdex-200 column (GE Healthcare) equilibrated with the running buffer containing 50 mM PBS pH 7.4, 150 mM NaCl by Akta purifier (GE Healthcare). Absorbance at 280 nm was detected. For the method of measuring protein concentration, we used Precision Red Advanced Protein Assay (Cytoskeleton) according to the manufacturer’s protocol.

### Mouse bacteremia models

Mouse bacteremia models was used essentially as described [11]. Outbred, immunocompetent female CD1 Swiss mice were 6 weeks of age at the time of use. We injected each mouse with 10^8^ CFUs of live *S. aureus* washed once with 0.1 mL PBS into the tail vein. Control animals received PBS only. After inoculation, mouse health and disease advancement were monitored every 8 h for up to 120 h. We euthanized the mice immediately if they showed signs of respiratory distress, mobility loss, or inability to eat and drink. All surviving animals were euthanized at 120 h.

The experimental procedures involving mice were carried out in strict accordance with the recommendations in the Guide for the Care and Use of Laboratory Animals of the National Institutes of Health (Beijing, China) and the State Key Laboratory of Pathogens and Biosecurity of the Institute of Microbiology and Epidemiology (Beijing, China). The protocol for animal handling and experimentation was approved by the Institutional Review Board of the Academy of Military Medical Science (AMMS, Beijing, China).

### Human blood survival assays

Human blood survival was assayed using a previously established protocol [26] with some changes. This experimental method was carried out in accordance with the approved guidelines of the Institutional Medical Ethics Committee of AMMS. *S. aureus* strains were grown to mid-exponential phase, washed once with sterile phosphate-buffered saline (PBS), then resuspended in PBS at 1×10^7^ CFUs/100 μL. 100 μL was added to 900 μL fresh blood samples obtained from healthy human volunteers. The tubes were incubated at 37°C with gentle rocking. At different time points, blood lysed by twice diluted 1% Saponin for 5 min on ice. Bacteria were then serially diluted in 10 fold dilutions in PBS and plated to determine the CFU/mL of viable bacteria. The percent survival was extrapolated to the original inoculum.

### Lysis of human neutrophils

Lysis of polymorphonuclear leukocytes (PMNs) by synthetic PSMs or aggregates was determined as described [11]. Human neutrophils (PMNs) were isolated from venous blood of healthy volunteers in accordance with neutrophil isolation kit (Sigma-Aldrich) protocols. The isolated PMNs were suspended in Hank’s balanced salt solution without Ca^2+^ and Mg^2+^ (HBSS, Gibco), and was identified by flow cytometry (Accuri C6). The purity of PMN was > 95%. Synthetic PSMs or aggregates were diluted in HBSS without Ca^2+^ and Mg^2+^, and were added to wells of a 96-well tissue culture plate containing 10^5^ PMNs and plates were incubated at 37°C for up to 30 min. At the desired times, PMN lysis was determined by lactate dehydrogenase (LDH) cytotoxicity detection kits (Promega) according to the manufacturer’s protocol.

### Transmission electron microscopy (TEM)

TEM was performed to visualize the fibrils and oligomers. Peptides or aggregates were dissolved in ultra-pure water to a concentration of 100 μg/mL. 10 μL samples were applied directly onto TEM grids with support films of Formvar/Carbon (Ted Pella), that were charged by high-voltage, alternating current glow-discharge, immediately before use. Grids were allowed to adhere for 10 min and negatively stained with 1.5% uranyl acetate for 30 s. Specimens were examined with a Hitachi HT7700 transmission electron microscope, at an accelerating voltage of 80 kV.

### Identification of peptides in-gel by liquid chromatography-tandem mass spectrometry (LC-TMS)

In-gel digestion was carried out by following a protocol [27] with slight modifications. In brief, purified products were run on a tricine-SDS-PAGE gel (Ezbiolab) and stained with eStain (Genscript). Corresponding protein bands were cut out. Gel pieces were washed with acetonitrile (Thermo Fisher Scientific) and 100 mM ammonium bicarbonate (Sigma) to decolor and dehydrate. Subsequently gel pieces digested with 5 μL trypsin (10 nM, Thermo Fisher Scientific) overnight. The next morning, enzymatic hydrolysis was halted with the addition of 5% formic acid to the acetonitrile solution, and then elute from the gel pieces by washing with acetonitrile. The peptides were dried using a vacuum centrifugal concentrator (miVac), dissolved in 0.1% formic acid in water, and used for mass spectrometry (MS) analyses.

For LC-TMS (also known as LC-MS/MS) analysis, peptides were separated by a 60 min gradient elution at a flow rate 0.3 μL/min with a Thermo-Dionex Ultimate 3000 HPLC system, which was directly interfaced with the Thermo Orbitrap Fusion mass spectrometer. The analytical column was a fused silica capillary column (75 μm ID, 150 mm length; Upchurch Scientific, Oak Harbor, WA) packed with C-18 resin (300 A, 5 μm; Varian, Lexington, MA). Mobile phase A consisted of 0.1% formic acid, and mobile phase B consisted of acetonitrile and 0.1% formic acid. The Orbitrap Fusion mass spectrometer was operated in the data-dependent acquisition mode using Xcalibur 3.0 software and there was a single full-scan mass spectrum in the Orbitrap (350–1550 m/z, 120,000 resolution) followed by 3 seconds data-dependent MS/MS scans in an Ion Routing Multipole at 30% normalized collision energy (HCD). The MS/MS spectra from each LC-MS/MS run were searched against the selected database using the Proteome Discoverer search algorithm(thermo proreome discoverver 2.1).

### High performance liquid chromatography (HPLC)

HPLC analysis was performed using the previously described method [28] or a new method established in this study. Chromatography was performed using a Kromasil 100-5-C18 (AkzoNobel) by Agilent 1100 series. Water/acetonitrile gradient in 0.1% trifluoroacetic acid from 50% to 100% acetonitrile for 30 min at a flow rate of 1 mL/min. Absorbance at 215 nm was detected using a photodiode array detector. The respective δ-toxin peaks were confirmed using peptide and MALDI/TOF-MS (see below).

### Matrix-assisted laser desorption/ionization time of flight mass spectrometry (MALDI-TOF-MS)

MALDI/TOF-MS analysis was performed using a new method established in this study with the technical support by the Tsinghua University (Beijing, China) Analysis Center. Sinapic acid (SA) was chosen as the MALDI matrix for oligomer detection, and α-Cyano-4-hydroxycinnamic acid (CHCA) was chosen as the MALDI matrix for peptide detection. The matrix solution was prepared by dissolving 20 mg 3-HPA and 45 mg dihydrogen ammonium citrate (DHAC) in a 1 mL mixture solution of acetonitrile/water (1:1, v/v). When testing an oligomer, we did not mix SA with the sample, but instead dried the three layers of SA-oligomer-SA on a steel plate to keep the original properties of the oligomer. MALDI-MS analysis was performed on AXIMA Performance (Shimadzu Scientific Instruments) using a standard stainless steel plate with manual pipetting. This instrument was equipped with a 337 nm nitrogen laser. The mass spectrum was acquired in the negative linear mode with an acceleration voltage of 20 kV, and the vacuum pressure in the ion source was (3–5) × 10^−6^ Torr. Data evaluation was done using the mass spectrometry software, UV Probe (Shimadzu Scientific Instruments).

### Cross-link assays

BS3 (bis(sulfosuccinimidyl)suberate) is an ammonium-based crosslinking agent. We mixed a 6 μL (1 mg/mL) sample with 3 μL BS3 (10 mM) in each reaction, and incubated it at 4°C for 30 minutes, then terminated the reaction with 1 μL DTT(1 M). The products were run on a tricine-SDS-PAGE gel (Ezbiolab) and stained with eStain (GenScript Biotech).

### Components of immunoprecipitation (Co-IP)

δ-Toxin antibody was purchased from Abgent. The PSMα antibody was induced by injection of a synthetic peptide into Balb/c mice, which was performed by 3 times subcutaneously and 2 times intraperitoneally. Polyclonal antibodies were purified from serum using a protein G column (GE Healthcare). Co-IP was performed using the Dynabeads Protein G Immunoprecipitation Kit (Thermo) according to the manufacturer’s protocol, with slight modifications. Each reaction system contained 200 μL, including 50 μL of magnetic beads, 10 μg of antibody, and 300 μg of peptides or aggregates, incubated at room temperature for 3 hours. The immunoprecipitation products were run on a tricine-SDS-PAGE gel (Ezbiolab) and stained with eStain (GenScript Biotech), and identified by LC-MS/MS.

### Nuclear Magnetic Resonance (NMR)

Measurements were taken at 298 K on a Bruker AVANCE II HD 600 MHz NMR spectrometer equipped with a cryoprobe. Peptide samples were obtained from purified products of ΔPSMα by HPLC. Samples (500 μL) of 1.5 mM fδ-toxin or dfδ-toxin were prepared in Methanol-d4. Nuclear Overhauser Effect Sectroscopy (NOESY) spectra were recorded with mixing times of 200 ms, and Total Correlation Spectroscopy (TOCSY) experiments were conducted with spin-lock times of 56 ms. The data sizes for the NOESY, TOCSY, and Double Quantum Filter Correlation Spectroscopy (DQF-COSY) experiments were 2048 (t1) × 512 (t2) points. The carrier frequency was centered on H_2_O resonance, and 1 H chemical shifts were referenced to water at 298 K (4.773 ppm). NMR data were processed and analyzed using NMRPipe [29] and Sparky [30]. Structure calculations were made using the Crystallography & NMR System (CNS) [31], with upper boundaries of 2.5, 3.0, and 5.0 Å and a lower boundary of 1.80 Å according to NOE intensity. 100 structures were calculated, from which the 10 structures with the lowest energy were selected. The calculated structures were evaluated using PROCHECK [32], PyMOL [33], and UCSF Chimera [34].

### Atomic Force Microscopy (AFM)

AFM assays were carried out by a protocol [35] with the following adjustments: Dilute the peptide to 400 nM with ultrapure water. Administer 80 μL of the diluted peptide onto a mica surfacetreated with (3-Aminopropyl)triethoxysilane (APTES). Let stand at room temperature for 30 min. Carefully draw off the upper layer of the solution. Rinse the surface of the mica gently with ultrapure water. Apply 80 μL ultrapure water. Then put the sample in a Multimode 8 AFM (Bruker) 10 × 10 μm E scanning tube, use ScanAsyst in fluid mode, and load the experiment using a silicon nitride (SNL-10) probe with an elastic coefficient of 0.12 N/m.

### Circular dichroism (CD) measurement

CD measurement was performed with technical support provided by Tsinghua University Protein Identification Platform. The structures of synthetic PSM peptides were analyzed by CD spectroscopy on a Chirascan-plus (Applied Photophysics) at room temperature. Solutions of δ-toxin peptides, each at 150 μg/mL, were prepared in ultrapure water. Measurements were performed in triplicate and the resulting scans were averaged and smoothed, and the buffer signal was subtracted.

### Microfluidic modulation spectroscopy (MMS)

MMS measurements were conducted using the automated AQS^3^ Pro system (RedshiftBio) with AQS^3^ analytics software [36]. Solutions of δ-toxin peptides, each at 2 mg/mL, were prepared in ultrapure water. All samples and their corresponding buffer blanks were preloaded into a 24-well plate in a pairwise manner. The samples and the buffers were degassed using a built-in well plate degasser for 30 min. An automated testing protocol, including all reference buffer and sample measurements, was set up in the acquisition software for each experiment in triplicate.

### Analytical ultracentrifugation (AUC)

AUC was performed in accordance with a previously published method [37], with some changes and technical support from the Tsinghua University Protein Identification Platform. Sedimentation experiments were carried out at 20°C in an XL-I analytical ultracentrifuge (Beckman–Coulter) equipped with Rayleigh Interference detection (655 nm). 400 μL sample (1 mg/mL) were centrifuged at 50,000 rpm for 8 h in an An-50 Ti rotor (Beckman Coulter) using 12 mm double-sector aluminum centerpieces. All samples were prepared in ultrapure water. Interference profiles were recorded every 6 min. Data analysis was conducted with the software program Sedfit 11.7. Theoretical sedimentation coefficients were calculated from the crystal structure PDB file using a Hydropro 7 c [38], with a hydrated radius of 3.1 Å for the atomic elements.

### Static light scattering (SLS)

SLS was performed using the previously described method [39] with technical support from the Tsinghua University Protein Identification Platform. 100 μL protein at 1 mg/mL was injected into a Superdex-200 column (GE Healthcare) equilibrated with running buffer comprised of 50 mM PBS at pH 7.4 and 150 mM NaCl. The chromatography system was coupled to an 18-angle light scattering detector (Wyatt Technology) for data collection. Data were collected every 0.5 s at a flow rate of 0.5 mL/min. Data analysis was done using the program ASTRA 6.1 (Wyatt Technology).

### Thioflavin T (ThT) fluorescence assay

ThT fluorescence assays were carried out by following a protocol [22] with slight modifications. The assays were performed in 96-well black, opaque, polystyrene, TC-treated plates (Corning). Each reaction system contained 200 μL, 50 μM peptide and 200 μM ThT in ultrapure water. Fluorescence curves were measured after slightly shaking by an SpectraMax i3 (Molecular Devices) at 438 nm excitation and 470–530 nm emission. For the fluorescence microscopy, the final concentrations for each reaction were 300 μM peptide and 50 μM ThT in ultrapure water. 2 μL of each sample were applied to a glass microscope slide and covered with a coverslip. The samples were then examined under an inverted fluorescence microscope (Olympus).

### Biofilm assay

Biofilm assays were determined as described [25,40], with some changes. Static biofilms were grown in 8-well Lab-Tek chambered cover glass plates (Thermo Fisher Scientific) for 48 h, peptides is added together with bacteria or added after 36 h of bacterial growth. PBS was used to gently wash away floating bacteria. Then PBS containing SDS (0.125%, m/v) was applied and allowed to soak for 5 minutes at room temperature. Thereafter additional PBS was used to wash away residual SDS. Treated biofilms were gently washed and stained with propidium iodide (10 μM) for 20 min. Images were taken with Fluoview 1000 (Olympus) confocal laser-scanning microscope (CLSM).

### Statistical analysis

Unless noted otherwise, statistical significance was assessed by analysis of variance (ANOVA) and indicated in the figure legends. The *P* values were calculated by Graph Pad Prism 8 software.

## Results

### *PSMs purified from* Staphylococcus aureus *(MW2) formed two different aggregates mainly composed of δ-toxin*

**Figure 1.**
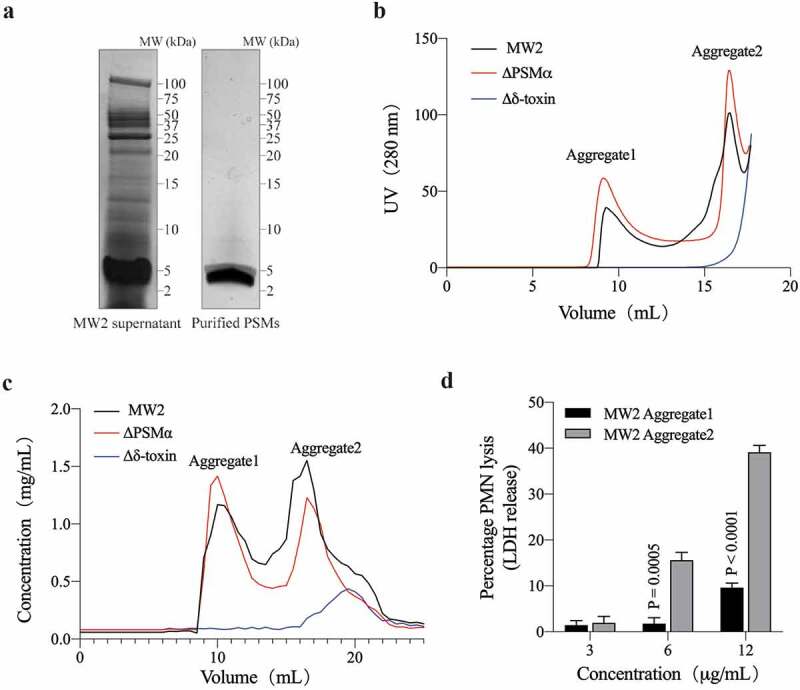
**PSM Aggregates from *Staphylococcus aureus* MW2 and its mutants**. (a) Tricine-SDS-PAGE of *S. aureus* (MW2) supernatant and the purified PMS product. (b) UV (280 nm) absorption curve of PSM purified from MW2 and its mutants by SEC analysis. There is a strong non-protein absorption peak around 20 mL, which is not included in the curve. (c) SEC result by measuring the protein concentration of each 0.5 mL fraction. (d) MW2 aggregate-induced lysis of human neutrophils as measured by released lactate dehydrogenase (LDH) activity at different concentrations. Data represent means ± SEM of at least three independent measurements. Statistical significance was determined by ANOVA following Sidak’s multiple-comparison test, the P-value is obtained by comparing Aggregate1 and Aggregate2 in the corresponding concentration. Experiments (a-c) were repeated independently three times with similar results

In our previous studies, we assumed that PSMs might exist to some extent in the form of aggregates in the supernatant of *S. aureus*. In order to obtain PSMs from CA-MRSA for further characterization of the aggregations, we used MW2 strains (USA400, ST1 lineage) which is the one of lineage of CA-MRSA isolates in the United States [41], to purify PSMs from MW2 supernatant (Figure 1(a)), and obtained two main aggregates by Size Exclusion Chromatography (SEC). The molecular weight (MW) of Aggregate1 was larger than 400 kDa, while Aggregate2 was about 20–30 kDa by the estimated retention volume and SEC standard products (Figure 1(b)). These results are much larger than the MW of any single PSM peptide [11]. Due to the lack of tryptophan and tyrosine in some PSMα peptides, their absorption of ultraviolet (UV) at 280 nm is weakened. Some non-protein impurities may also distort the absorption of UV, so we measured protein concentration with all the components of SEC to revise its absorption curve (Figure 1(c)). By liquid chromatography-tandem mass spectrometry (LC-MS/MS) analysis, Aggregate1 is composed of δ-toxin, and Aggregate2 includes δ-toxin and PSMα1-4 (Table 1). Importantly, Aggregate2 showed a much stronger ability to lyse neutrophils compared to that of Aggregate1 (Figure 1(d)). This is very likely due to Aggregate2 containing PSMα peptides which have high cytolytic capacity. SEC results from the PSMα and δ-toxin deletion mutants showed that the PSMα mutant (ΔPSMα) still formed Aggregate2 of the same size, but the δ-toxin mutant (Δδ-toxin) did not form any similar aggregates (Figure 1(b-c)). This suggests that δ-toxin is not only a component of Aggregate1, but also the main and indispensable component of Aggregate2.

**Table 1.** Identification of MW2 aggregates by LC-MS/MS

Sample	Accession	Description	Peptides	PSMs	Unique Peptides	Coverage	Score Sequest HT
Aggregate1	WP_001823225.1	δ-toxin	2	28	2	46	65.1906496
Aggregate2	WP_001823225.1	δ-toxin	3	55	3	100	123.259218
	WP_014373781.1	PSMα1	3	4	2	100	9.06406713
	WP_014373780.1	PSMα2	2	3	1	85.7	7.3963623
	WP_014373779.1	PSMα3	4	6	4	72.3	4.26563871
	WP_014532416.1	PSMα4	2	4	2	60	8.73086214

PSM, peptide spectrum match. Coverage, coverage of peptides on the protein. Peptide, number of matched peptide types. Unique peptides, number of matched specific peptide types. Score Sequest HT, protein match score.

### Aggregate1 forms fibrils, while Aggregate2 forms oligomers

**Figure 2.**
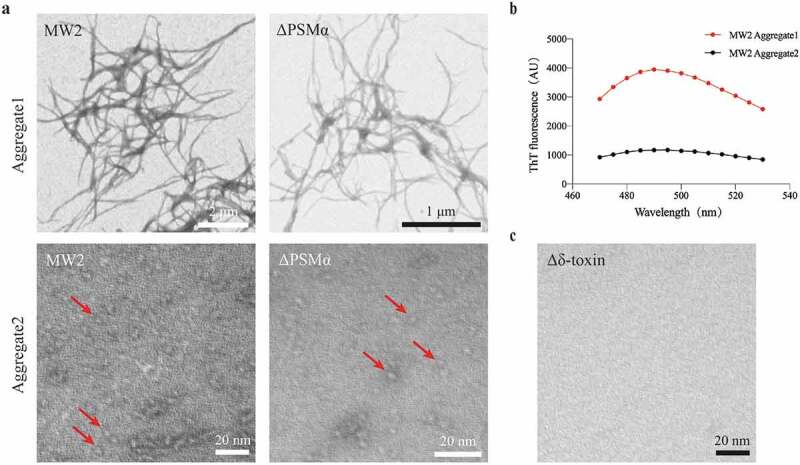
**TEM micrographs of PSM aggregates**. (a) TEM micrographs of MW2 and ΔPSMα aggregates. The image scale has been marked in bar length in the lower right corner of the image. Arrows (red) in the images point to the locations of oligomers. (b) MW2 Aggregate1 displays a marked ThT fluorescence peak around 490 nm as compared with the fluorescence peak of MW2 Aggregate2. (c) TEM micrographs of Δδ-toxin do not display any attributes characteristic of aggregates. Experiments in (a-c) were repeated independently three times with similar results

TEM imaging of the aggregates revealed that the Aggregate1 has obvious amyloid fibrils, which are very similar to the *S. aureus* functional amyloids in the study of Schwartz K *et al*. [40]. The Aggregate1 fibrils also bound the amyloid-indicator dye ThT, generating high levels of fluorescence at an emission spike near 490 nm and a characteristic amyloid-fibrillation curve. Aggregate2 is globular, a few nanometers in diameter, and does not bind ThT with no fluorescence emissions correspondingly. (Figure 2(a-b)). These findings combined with the results of SEC (Figure 1(a-b)), indicate that these globular proteins may be oligomers consisting of several peptides. Soluble oligomers are common to most amyloids, like the amyloid-β (Aβ) peptide in Alzheimer’s disease [42]. In addition, TEM imaging of aggregates from the MW2 and ΔPSMα strains also maintain high consistency (Figure 2(a)), and Δδ-toxin don’t have any aggregates (Figure 2(c)). Therefore, for both MW2 and ΔPSMα strains, Aggregate 1 is composed of δ-toxin fibrils, but Aggregate2 oligomers from MW2 are composed of δ-toxin and PSMα, and Aggregate2 oligomers from ΔPSMα are composed of δ-toxin only.

### ΔPSMα Aggregate2 is an 8 monomer oligomer composed of δ-toxin

**Figure 3.**
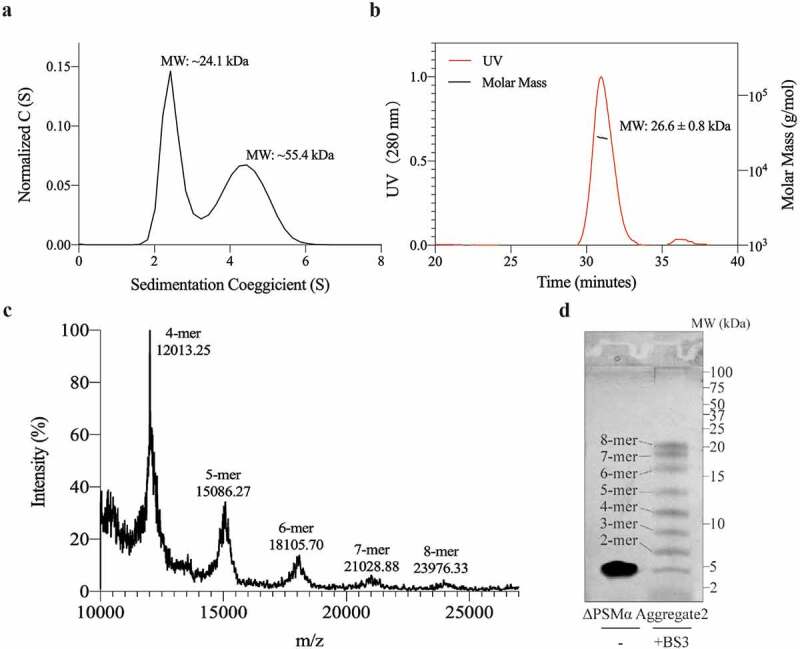
**ΔPSMα Aggregate2 is composed of an 8-mer oligomer**. (a) Results of analytical ultracentrifugation on ΔPSMα Aggregate2. The curve is the sedimentation coefficient distribution plot. The molecular weight was calculated using Sedfit 11.7 software. (b) Static light scattering (SLS) study of ΔPSMα Aggregate2. The molecular weight was calculated using ASTRA 6.1, and is shown beside the peak. (c) ΔPSMα Aggregate2 is analyzed in the native MALDI-TOF-MS with a mass-to-charge ratio (m/z). The estimated number of monomers and the molecular mass are shown above the peaks. (d) Tricine-SDS-PAGE shows the cross-link assay results of ΔPSMα Aggregate2 enhanced by the crosslinker BS3. The number of monomers are shown in the images. All the experiments were repeated independently three times with similar results

In order to determine the properties of Aggregate2, we first analyzed the ΔPSMα Aggregate2, which only be composed of δ-toxin. Generally, the oligomer formed by α-helix peptides like Aβ_42_ is very unstable and likely to undergo conversion between monomers and various complex oligomer species [43,44]. However, Aggregate2 can remain stable at a temperature of 4°C for several weeks or even longer. We used static light scattering (SLS) and analytical ultracentrifugation (AUC) to obtain more accurate MW information than SEC(Figure 3(a-b)). These results showed that its MW is about 24 kDa, while the MW of a δ-toxin monomer is about 3 kDa. Correspondingly, we found ~24 kDa peaks from native matrix-assisted laser desorption/ionization time of flight mass spectrometry (MALDI-TOF-MS) experiments, and eight bands were obtained from the treatment of BS3 cross-linking agent (Figure 3(c-d)). Therefore, the Aggregate2 of ΔPSMα is an 8 monomer oligomer composed of δ-toxin.

### MW2 Aggregate2 is an oligomer complex composed of δ-toxin and PSMα

**Figure 4.**
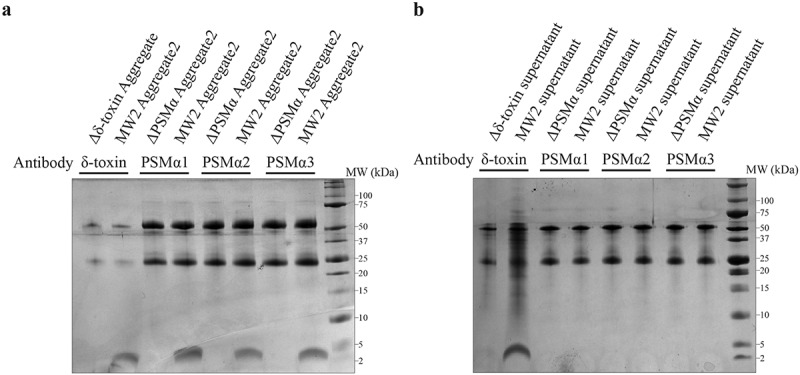
**Tricine-SDS-PAGE analysis of the components of co-immunoprecipitation (Co-IP)**. (a-b) The images of Tricine-SDS-PAGE stained by Coomassie Blue. The upper two bands in the gel are antibodies, the lower band consists of Co-IP protein products. The band components have been digested in-gel and identified by LC-MS/MS. (a-b) correspond to different samples: (a) is Aggregate2 from MW2 and its mutants, (b) is the supernatant of MW2 and its mutants. Perhaps due to the affinity of the PSMα antibody, we could not obtain a sufficient amount of product in supernatant Co-IP assays (b). All the experiments were repeated independently three times with similar results

In contrast to the ΔPSMα strain, in which Aggregate2 is composed of a single δ-toxin, the Aggregate2 of MW2 strain is much more complicated. From the results of SEC (Figure 1(b-c)), we speculate that PSMα combines with δ-toxin so that PSMα (~3 kDa) appears in the Aggregate2 (20–30 kDa). In order to verify our conjecture, we used the polyclonal antibodies of δ-toxin, PSMα1, PSMα2, and PSMα3 to bind with magnetic beads and incubate with Aggregate2 of MW2 (we did not obtain usable PSMα4 antibody), and then use LC-MS/MS to determine the components of immunoprecipitation (Co-IP) products to ascertain whether the δ-toxin and PSMα are bound. The results are not surprising: when the control shows that none of the antibodies have nonspecific binding, regardless of which antibody, the Co-IP products include all components of δ-toxin and PSMα1-4 (Figure 4(a), Table 2), which shows that PSMα1-4 and δ-toxin combined with each other in Aggregate2 of MW2. Thus, MW2 Aggregate2 is an oligomer complex. Likewise, we got similar Co-IP results when we directly incubated in the MW2 supernatant (Figure 4(b), Table 2). These results represent the combination of PSMα and δ-toxin also in the MW2 supernatant. PSMα and δ-toxin are both α-helical amphiphilic peptides, they may bind to each other through hydrophobic interactions [45]. However, the size of their complexes maintained a particular consistency, which suggests that they are organized aggregation, rather than completely random behavior.

**Table 2.** Identification of Co-IP results by LC-MS/MS

Sample	Antibody	Accession	Description	Peptides	PSMs	Unique Peptides	Coverage	Score Sequest HT
MW2 Aggregate2	δ-toxin	WP_001823225.1	δ-toxin	2	6	2	46	22.0601952
		WP_014373781.1	PSMα1	4	9	3	100	21.1979103
		WP_014373780.1	PSMα2	5	9	4	100	23.0848207
		WP_014373779.1	PSMα3	5	7	5	100	13.8474682
		WP_014532416.1	PSMα4	3	11	3	100	31.6470328
MW2 Aggregate2	PSMα1	WP_001823225.1	δ-toxin	3	7	3	100	23.9936047
		WP_014373781.1	PSMα1	4	12	3	100	31.0159116
		WP_014373780.1	PSMα2	4	8	3	100	22.4521281
		WP_014373779.1	PSMα3	4	7	4	100	15.6767581
		WP_014532416.1	PSMα4	3	16	3	100	39.6538506
MW2 Aggregate2	PSMα2	WP_001823225.1	δ-toxin	3	10	3	100	31.4363673
		WP_014373781.1	PSMα1	3	12	2	100	31.4386984
		WP_014373780.1	PSMα2	5	13	4	100	35.3863463
		WP_014373779.1	PSMα3	7	18	7	100	46.5289361
		WP_014532416.1	PSMα4	3	8	3	100	19.7028577
MW2 Aggregate2	PSMα3	WP_001823225.1	δ-toxin	3	8	3	100	18.6013126
		WP_014373781.1	PSMα1	4	10	3	100	21.5403515
		WP_014373780.1	PSMα2	5	10	4	100	27.9978607
		WP_014373779.1	PSMα3	5	10	5	100	23.9588108
		WP_014532416.1	PSMα4	3	16	3	100	47.1002944
MW2 supernatant	δ-toxin	WP_001823225.1	δ-toxin	2	4	2	46	10.6424484
		WP_014373781.1	PSMα1	4	6	3	100	16.4154882
		WP_014373780.1	PSMα2	3	4	2	100	9.61245632
		WP_014373779.1	PSMα3	3	4	3	100	11.4299357
		WP_014532416.1	PSMα4	3	8	3	100	17.0296268

PSM, peptide spectrum match. Coverage, coverage of peptides on the protein. Peptide, number of matched peptide types. Unique peptides, number of matched specific peptide types. Score Sequest HT, protein match score.

### fδ-Toxin tends to form fibrils, while dfδ-toxin tends to form oligomers

**Figure 5.**
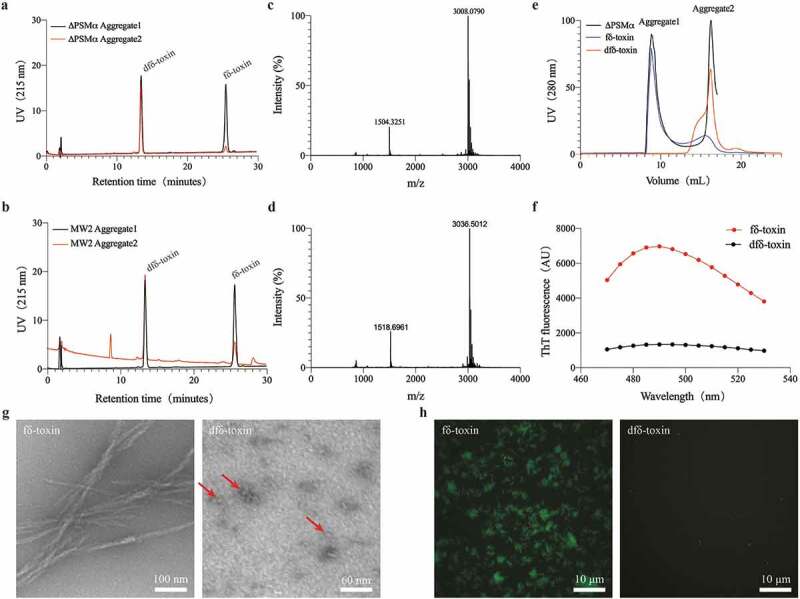
**The two aggregates are mainly composed of different δ-toxins**. (a, b) HPLC analyzation of MW2 and ΔPSMα, respectively. The labeling of the components is derived from the MALDI identification results. (c-d) MALDI-TOF-MS identifies the results of HPLC peaks. (c) The identification results of HPLC peaks with a retension time of ~14 min. The MW of dfδ-toxin is ~3008 Da. (d) The identification result of HPLC peaks corresponding to a retention time of ~26 min. The MW of fδ-toxin is ~3036 Da. (e) UV (280 nm) absorption curve of two δ-toxins in SEC analysis compared to ΔPSMα. (g) TEM micrographs of two δ-toxins. Image scale bars are included in the lower right corner of the images. The arrows (red) in the images point to the locations of oligomers. (f, h) Thioflavin T (ThT) fluorescence curve and images of two δ-toxins, respectively. In (h), image scale bars are located in the lower right corner of the images. All the experiments were repeated independently three times with similar results

In the ΔPSMα strain, because δ-toxin formed two relatively stable aggregates (fibril and oligomer) in the same condition, we speculated that the composition of the two aggregates should not be exactly the same. Unsurprisingly, in the analysis of HPLC combined with MALDI-TOF-MS, we found that Aggregate1 of the ΔPSMα was mostly composed of fδ-toxin (more than 50%), while Aggregate2 of the ΔPSMα was mostly composed of dfδ-toxin (more than 85%). Similarly, we obtained analogous results in the MW2 strain (Figure 5(a-d)). We speculated that fδ-toxin tends to form fibrils, while dfδ-toxin tends to form oligomers. We then used synthetic peptides to evaluate our hypothesis. According to the results of SEC, TEM, and ThT-staining, fδ-toxin showed similarity to Aggregate1 as amyloid fibrils, while dδ-toxin showed similarity to Aggregate2 as oligomer (Figure 5(e-h)). Therefore, the difference between the two aggregates is derived from the two different forms of δ-toxin. Aggregate1 is fibrillar due to fδ-toxin, and Aggregate2 is oligomeric on account of dfδ-toxin.

### Three-dimensional structure of fδ-toxin and dfδ-toxin in methanol solution

**Figure 6.**
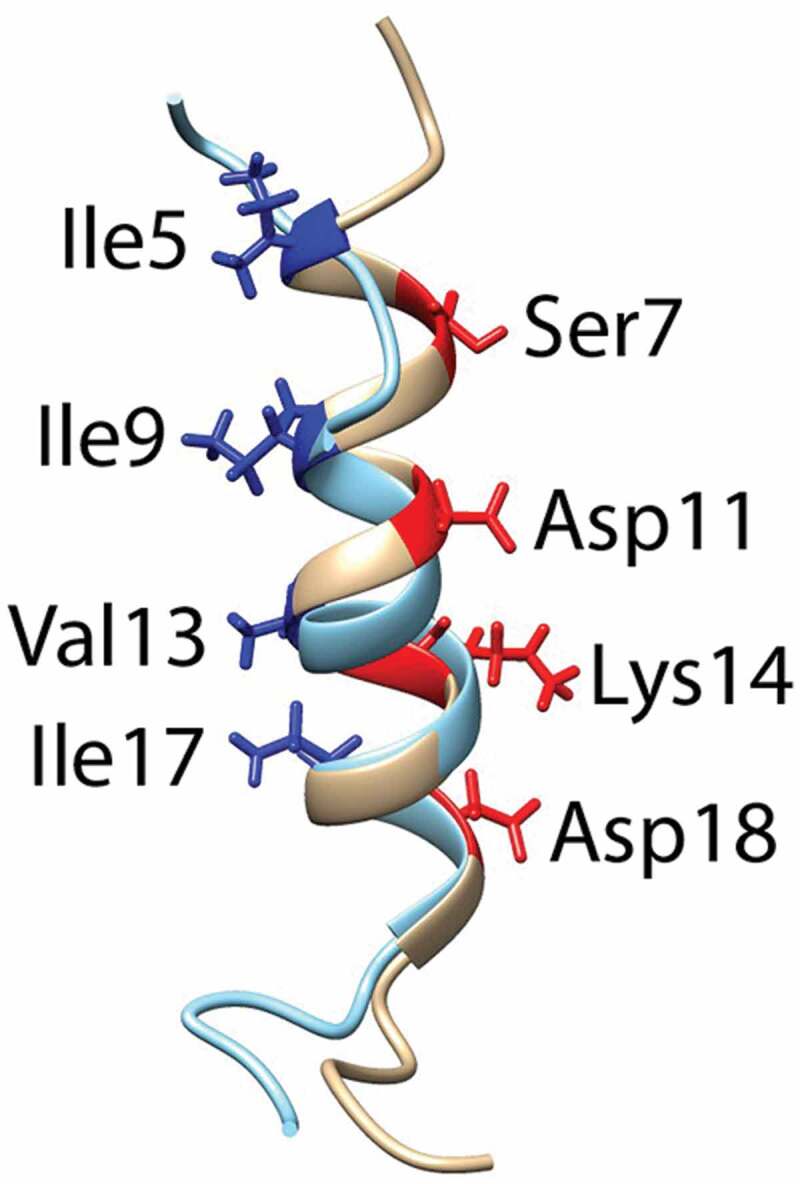
Superimpostion of the NMR structures of dfδ-toxin (gray) and fδ-toxin (cyan) in ribbon mode, with the side chains on the hydrophilic (red) and hydrophobic (blue) sides shown, and the corresponding residues labeled

fδ-Toxin and dfδ-toxin have the same sequence, but whether there is an N-terminus formyl modification causes the difference in aggregate. We used NMR to analyze the three-dimensional structure of the two monomer peptides in methanol, to try to compare their differences. The structures of dfδ-toxin and fδ-toxin are largely helical. In dfδ-toxin, a typical amphipathic helical structure extends from residue 5 to 20, with a hydrophilic side formed mainly by the side chains of Ser7, Asp11, Lys14, and Asp18, and a hydrophobic side formed mainly by Ile5, Ile9, Val13, and Ile17. While in fδ-toxin, the helicity extends over a shorter range, from residue 9 to 20, indicating that the N-formylation hampers the helical conformation (Figure 6, Table 3). We speculate that the difference in the secondary structure of peptides directly leads to the difference in its aggregation ability, but we have not found enough evidence or published reference data for how the formylation affects their secondary structure. This determination still requires further structural studies.

**Table 3.** Structural statistics for the family of 10 lowest energy structures

	dfδ-toxin	fδ-toxin
(a) Distance restraints		
Intraresidue	146	182
Sequential (|i − j| = 1)	74	53
Medium-range (2 ≤ |i − j| ≤ 4)	60	62
Long-range (|i − j| ≥ 5)	0	0
Total	280	297
(b) Atomic r.m.s. differences (Å)^a^; residues 9–20		
Backbone heavy atom (C_α_)	0.182 ± 0.068	0.421 ± 0.171
Heavy atoms	0.604 ± 0.056	0.753 ± 0.168
(c) Ramachandran plot^b^ (% residues)		
Residues in most favored regions	51.7	62.5
Residues in additional allowed regions	39.2	32.1
Residues in generously allowed regions	5.4	3.3
Residues in disallowed regions	3.8	2.1

^a^The precision of the atomic coordinates is defined as the average r.m.s. difference between the 10 final structures and the mean coordinates of the protein.

^b^The program Procheck was used to assess the overall quality of the structures.

### fδ-Toxin oligomers tend to assemble into fibrils, while dfδ-toxin oligomers remain isolated in solution

**Figure 7.**
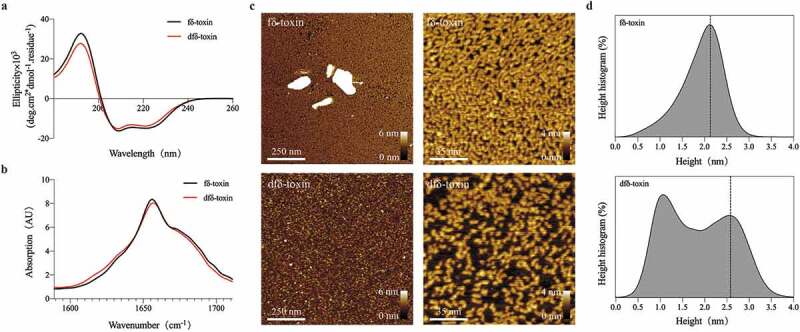
**The state of two δ-toxins in aqueous solution**. (a) Circular dichroism (CD) measurement of two δ-toxins at room temperature. Both δ-toxins show the characteristic curve of an α-helix, as determined by CDNN software. Measurements were performed in triplicate and the resulting scans were averaged, smoothed, and the buffer signal was subtracted. (b) MMS measurement of two δ-toxins at room temperature. The bands between 1654 and 1658 cm^−1^ is consistent with the dominant α-helix [64]. Measurements were performed in triplicate and the resulting scans were averaged, smoothed, and the buffer signal was subtracted. (c) AFM visualization of two δ-toxin aggregates. The image and height scales are marked in the images. (d) The curve shows the height statistics of the two δ-toxin aggregates, as computed using the visualization program Gwyddion. All the experiments were repeated independently three times with similar results

Our results show that after dfδ-toxin and fδ-toxin are dissolved in water, they already exhibit different polymerization states without any manipulation and incubation (Figure 5(e-h)). To understand the difference in aggregation ability between fδ-toxin and dfδ-toxin, we measured the secondary structure of fδ-toxin fibrils and dfδ-toxin oligomers in aqueous solution, and found that they both also contain α-helices (Figure 7(a-b)). We speculated that they form cross-α architectures similar to PSMα3 fibrils [22], which would be consistent with the structure predicted in a previous study [46]. In addition, we observed fδ-toxin and dfδ-toxin in aqueous solution using Atomic Force Microscopy (AFM). Both fδ-toxin and dfδ-toxin had oligomer particles in the solution. The difference was that the fδ-toxin oligomers were slightly smaller (~2.1 nm), and could connect to form small short fibrils, while the dfδ-toxin oligomer was larger (~2.6 nm) and don’t combined (Figure 7(c-d)). Consequently, we speculate that their secondary structures lead to the differences oligomer: fδ-toxin oligomers can continue to assemble into fibrils, but dfδ-toxin oligomers will remain in an oligomeric state. So, fδ-toxin tends to form fibrils, while dfδ-toxin tends to form oligomers.

### fδ-Toxin fibrils enhance the stability of biofilms, while dfδ-toxin oligomers promote dispersal

**Figure 8.**
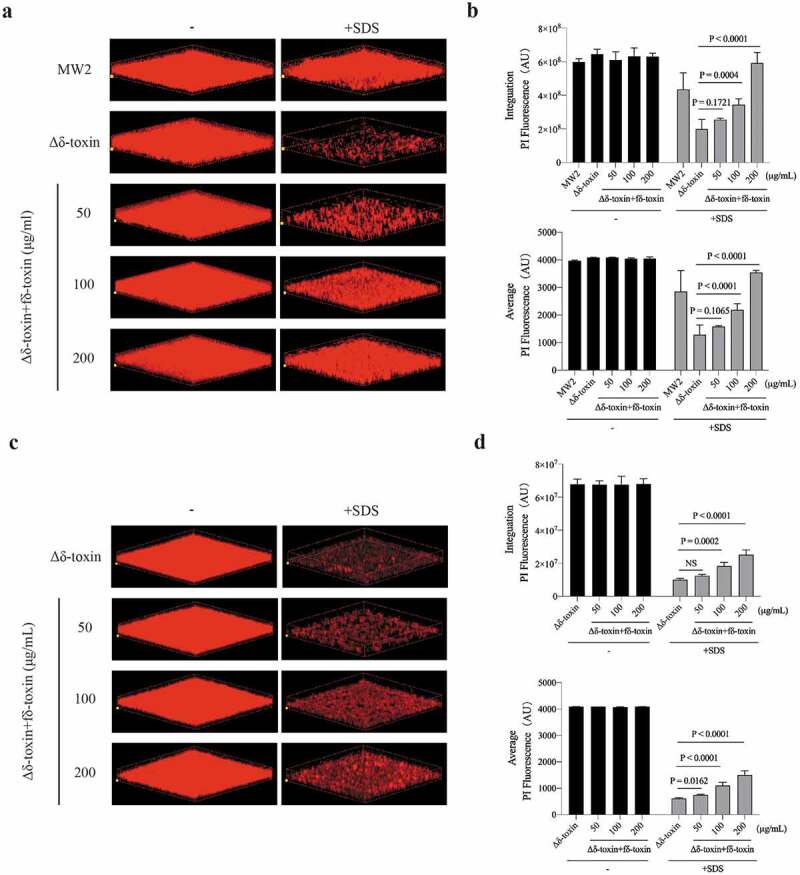
**Impact of adding fδ-toxin fibrils on the structure of static *S. aureus* biofilms**. Static biofilms were grown in eight-well chambered coverglass plates for 48 h, with adding fδ-toxin fibrils (a, b) before the formation of the biofilm (0 h) or (c, d) after the formation of the biofilm (36 h), and to test the dispersal mediated by SDS. (a, c) Three-dimensional confocal laser scanning microscopy (CLSM) images of biofilms. Extensions and scale are the same in every image (total *x* extension: 160 μm; total *y* extension: 160 μm). (b, d) Biofilm parameters were measured in at least 5 randomly chosen biofilm CLSM images of the same extension on a Fluoview 1000. Data represent means ± SD. ANOVA was used to determine statistical significance followed by Dunnett’s multiple comparison test. All the experiments were repeated independently three times with similar results

**Figure 9.**
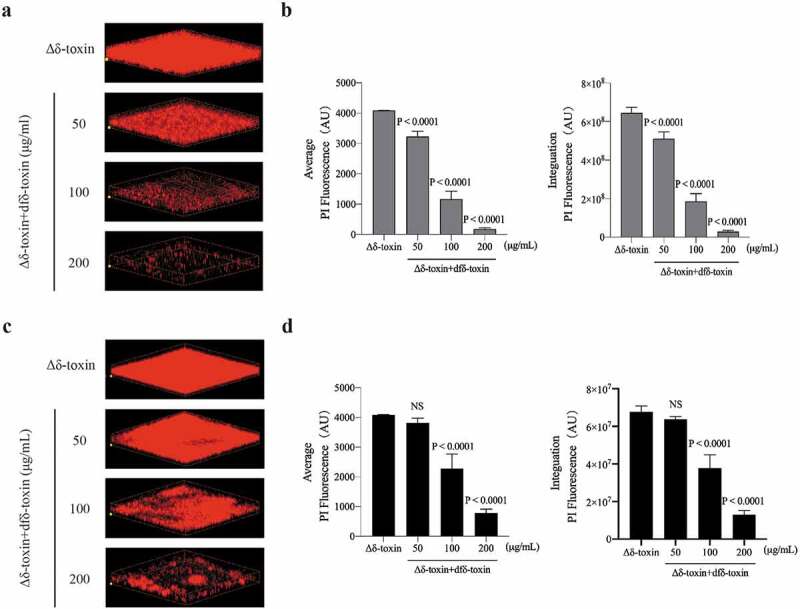
**Impact of adding dfδ-toxin oligomer on the structure of static *S. aureus* biofilms**. Static biofilms were grown in eight-well chambered coverglass plates for 48 h, with adding dfδ-toxin oligomer (a, b) before the formation of the biofilm (0 h) or (c, d) after the formation of the biofilm (36 h). (a, c) Three-dimensional confocal laser scanning microscopy (CLSM) images of biofilms. Extensions and scale are the same in every image (total *x* extension: 160 μm; total *y* extension: 160 μm). (b, d) Biofilm parameters were measured in at least 5 randomly chosen biofilm CLSM images of the same extension on a Fluoview 1000. Data represent means ± SD. ANOVA was used to determine statistical significance followed by Dunnett’s multiple comparison test. All the experiments were repeated independently three times with similar results

PSMs act as biofilm structuring factors in the biofilm-forming pathogen *S. aureus*, which impacts biofilm structure, detachment, and *in vivo* dissemination [25,47]. Of note, Schwartz K *et al*. posited that PSMs can modulate biofilms: soluble PSMα1 assist biofilm disassembly, while PSM fibrils resist biofilm dispersal [40]. In order to observe whether fδ-toxin fibrils and dfδ-toxin oligomers have a similar regulatory effect in biofilms, we examined biofilm structure in detail using high-resolution imaging of biofilm with confocal laser-scanning microscopy (CLSM). The biofilm formed by the Δδ-toxin strain was severely damaged under the treatment of surfactants (sodium dodecyl sulfate, SDS), while the MW2 strain showed great resistance. Whether adding fδ-toxin fibrils before or after biofilm formation, the biofilm of the Δδ-toxin both showed good resistance to SDS (Figure 8). On the contrary, when adding dfδ-toxin before or after biofilm formation, the biofilm of Δδ-toxin disassembled (Figure 9), even without the application of SDS. Thus, these results provide evidence that δ-toxin is involved in the regulation of *S. aureus* biofilms: fδ-toxin fibrils resist biofilm dispersal, while dfδ-toxin oligomers assist biofilm disassembly.

### The dfδ-toxin and PSMα oligomer complex had greater cytolytic function than PSMα alone

**Figure 10.**
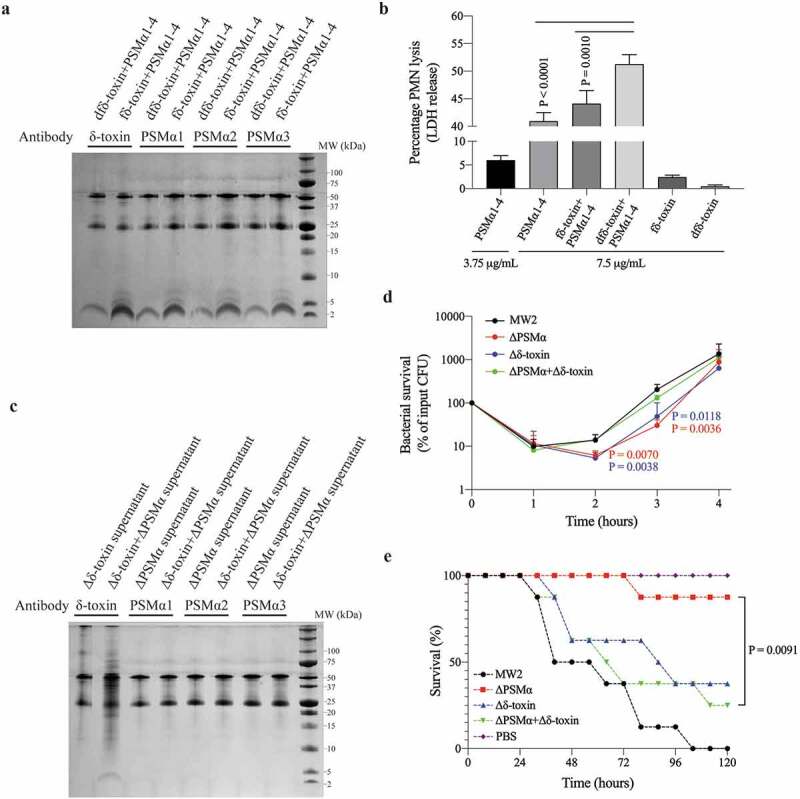
**The complex of δ-toxin and PSMα had greater cytolytic function than PSMα alone**. (a, c) The images of Tricine-SDS-PAGE stained by Coomassie Blue. The upper two bands in the gel are antibodies, the lower band consists of Co-IP protein products. The band components have been digested in-gel and identified by LC-MS/MS. (a,c) correspond to different samples: (a) is a mixture of synthetic peptides. (c) is the supernatant of mixture strain of ΔPSMα and Δδ-toxin. (b) Mixture of synthetic peptides induced lysis of human neutrophils as measured by the activity of released lactate dehydrogenase (LDH) in different concentrations. Data represent means ± SEM of at least three independent measurements. Statistical significance was determined by ANOVA followed by Dunnett’s multiple comparison test. (d) Survival curve of MW2 and its mutants in human blood. 10^7^ CFUs of live strains were inoculated into freshly drawn human blood and incubated for 4 h at 37°C. Bacterial survival was measured by counting CFUs every hour. Data represent means ± SD of 4 independent donors. Statistical significance was determined using ANOVA followed by Dunnett’s multiple comparison test. The P-value was obtained by comparison with Δδ-toxin+ΔPSMα at the corresponding time. (e) Bacteremia model survival curve. 10^8^ CFUs of live MW2 or its mutants or mixed strains in 0.1 mL PBS were injected into the tail veins of female CD1 Swiss mice (n = 8). Control animals received blank PBS buffer. Statistical analysis was performed using the Kaplan-Meier test for survival curves. Experiments were repeated independently three times with similar results

We mixed synthetic peptides (dfδ-toxin: PSMα1-4 = 1:1, m:m) to simulate the complex, and the combination of dfδ-toxin and PSMα was verified by Co-IP (Figure 10(a), Table 4). We conducted neutrophil lysis assays in which synthetic fδ-toxin, dfδ-toxin, and PSMα1–4 were combined in solution in followed by addition of the analyte neutrophils. The combination of dfδ-toxin and PSMα (dfδ-toxin: PSMα1-4 = 1:1, m:m) lysed many more neutrophils than any of the other tested combinations (Figure 10(b)). Thus, The dfδ-toxin and PSMα oligomer complex had greater cytolytic function than PSMα alone.

**Table 4.** Identification of Co-IP results by LC-MS/MS

Sample	Antibody	Accession	Description	Peptides	PSMs	Unique Peptides	Coverage	Score Sequest HT
fδ-toxin+ PSMα1-4	δ-toxin	WP_001823225.1	δ-toxin	3	88	3	46	184.803073
		WP_014373781.1	PSMα1	2	40	1	85.7	66.5116254
		WP_014373780.1	PSMα2	2	8	1	85.7	11.1160526
		WP_014373779.1	PSMα3	2	21	2	59	19.7084937
		WP_014532416.1	PSMα4	3	46	3	100	65.908283
fδ-toxin+ PSMα1-4	PSMα1	WP_001823225.1	δ-toxin	2	24	2	46	33.563807
		WP_014373781.1	PSMα1	3	90	2	100	146.496155
		WP_014373780.1	PSMα2	3	60	2	100	100.924703
		WP_014373779.1	PSMα3	2	44	2	59	61.4214191
		WP_014532416.1	PSMα4	3	62	3	100	125.680256
fδ-toxin+ PSMα1-4	PSMα2	WP_001823225.1	δ-toxin	3	60	3	46	115.487663
		WP_014373781.1	PSMα1	2	47	1	85	72.6561546
		WP_014373780.1	PSMα2	3	13	2	100	14.5863985
		WP_014373779.1	PSMα3	3	17	3	72.7	15.5047214
		WP_014532416.1	PSMα4	3	40	3	100	32.7210861
fδ-toxin+ PSMα1-4	PSMα3	WP_001823225.1	δ-toxin	2	43	2	46	75.2731302
		WP_014373781.1	PSMα1	2	44	1	85.7	66.3551342
		WP_014373780.1	PSMα2	2	8	1	85.7	7.80959654
		WP_014373779.1	PSMα3	2	12	2	59	10.5193903
		WP_014532416.1	PSMα4	3	35	3	100	28.0506523
dfδ-toxin+ PSMα1-4	δ-toxin	WP_001823225.1	δ-toxin	2	36	2	46	59.4873036
		WP_014373781.1	PSMα1	1	32	1	42.8	34.0332463
		WP_014373780.1	PSMα2	2	3	1	57.1	5.73431206
		WP_014373779.1	PSMα3	2	2	2	59	3.25767004
		WP_014532416.1	PSMα4	2	9	2	85	7.35288882
dfδ-toxin+ PSMα1-4	PSMα1	WP_001823225.1	δ-toxin	3	89	3	46	146.865549
		WP_014373781.1	PSMα1	2	86	1	85.7	113.777446
		WP_014373780.1	PSMα2	3	15	2	100	22.8838329
		WP_014373779.1	PSMα3	2	33	2	59	23.749366
		WP_014532416.1	PSMα4	3	75	3	100	91.7607554
dfδ-toxin+ PSMα1-4	PSMα2	WP_001823225.1	δ-toxin	2	43	2	46	63.665399
		WP_014373781.1	PSMα1	1	31	1	42.8	37.7084906
		WP_014373780.1	PSMα2	3	4	2	100	6.07426846
		WP_014373779.1	PSMα3	2	4	2	59	1.62622821
		WP_014532416.1	PSMα4	2	25	2	85	34.1270549
dfδ-toxin+ PSMα1-4	PSMα3	WP_001823225.1	δ-toxin	2	35	2	46	59.025749
		WP_014373781.1	PSMα1	1	34	1	42.8	59.7072719
		WP_014373780.1	PSMα2	4	5	3	100	9.23456597
		WP_014373779.1	PSMα3	2	7	2	59	5.80549681
		WP_014532416.1	PSMα4	3	21	3	100	27.0792433
Δδ-toxin +ΔPSMα supernatant	δ-toxin	WP_001823225.1	δ-toxin	3	32	3	46	90.8189472
		WP_014373781.1	PSMα1	4	32	3	100	84.1499232
		WP_014373780.1	PSMα2	5	22	4	100	59.9992867
		WP_014373779.1	PSMα3	8	30	8	100	80.7276129
		WP_014532416.1	PSMα4	3	17	3	100	48.9647826

PSM, peptide spectrum match. Coverage, coverage of peptides on the protein. Peptide, number of matched peptide types. Unique peptides, number of matched specific peptide types. Score Sequest HT, protein match score.

By determine the Co-IP products, we have verified that the supernatant of the MW2 cells or the mixture cells (Δδ-toxin: ΔPSMα = 1:1, CFU) would contain δ-toxin and PSMα in complex(Figure 4(b), Figure 10(c), Tables 2, 4), whereas the Δδ-toxin and ΔPSMα could not contain such complexes. In the pathogenesis of *S. aureus*, an important role of PSMs is allowing ingested bacteria to escape after neutrophilic phagocytosis by promoting the lysis of the engulfing neutrophils [48]. Therefore, it seems possible that bacteria cells with complex comprising dfδ-toxin and PSMα may have a greater capacity to help cells survive than cells lacking such complex. Apparently supporting this, when we assayed *S. aureus* survival in human blood with the MW2 strain, the ΔPSMα strain, the Δδ-toxin strain, or a 1:1 mixture of the ΔPSMα and Δδ-toxin strains. We found the cells of the ΔPSMα and Δδ-toxin strain were the least viable, and then mixture cells and MW2 cells (Figure 10(d)). This suggests that the presence of the dfδ-toxin and PSMα also somehow help *S. aureus* cells survive in human blood.

Pursing the potential biomedical relevance of these insights, we also exposed mice to four different bacterial challenges: the MW2 strain, the ΔPSMα strain, the Δδ-toxin strain, or a 1:1 mixture of the ΔPSMα and Δδ-toxin strains. We found that the MW2 strain challenge was the most deadly to the infected mice, followed in decreasing order by the combined strain challenge, the Δδ-toxin strain, and the ΔPSMα strain (Figure 10(e)). Thus, the presence of both Δδ-toxin and ΔPSMα apparently enables the formation of an dfδ-toxin and PSMα complex which renders *S. aureus* relatively more deadly to mice.

## Discussion

δ-Toxin is secreted in very large quantities in many strains of *S. aureus*, but researchers have not been able to discern which of its functions requires such large secretions, even though it has been studied for many years. Although superficially it has the appearance of an antibacterial peptide, δ-toxin has only shown limited antibacterial ability [15]. The fδ-toxin fibril plays an important role in the stability of its biofilm. Studies have found that the aggregate performance of δ-toxin has an important relationship with its concentration [49]. The extracellular DNA (eDNA) produced by bacteria may act as an attractant to δ-toxin to increase the local concentration and promote the process of fibril assembly [50]. Interestingly, Δδ-toxin increased the amount of cell surface PSMα, and decreased the amount of culture supernatant PSMα [28]. Combined with our research, we speculated that the amphiphilic PSMα peptides tend to adhere to the surface of bacteria rather than to remain in the cell culture supernatant. Combined with PSMα, dfδ-toxin oligomers transport PSMα from the cell surface into the culture supernatant, simultaneously, enhancing the function of PSMα. Therefore, although dfδ-toxin does not have noticeable features, it may be a powerful auxiliary for *S. aureus*, helping PSMα to perform its functions, such as cytotoxity. In addition, in our Co-IP experiments on *S. aureus* supernatants, we found that δ-toxin is not only bound to PSMα, but also to some proteins of other sizes (Figure 4(b)), indicating that δ-toxin may have a similar transporting interaction with several other exotoxins. For example, PSMs are required for mobilizing lipoproteins, the TLR2 agonists, from the *S. aureus* cytoplasmic membrane, but δ-toxin mutant had limited effect in lipoprotein shedding [51].

Our research shows differences between the functions of fδ-toxin and dfδ-toxin. The fδ-toxin fibrils correspond to biofilm stability to resist host or drug killing, while the oligomers of dfδ-toxin correspond to the dispersion of biofilms, and enhance the function of PSMα to achieve expansion. *S. aureus* translation of δ-toxin is initiated with formyl-methionine, but the N-terminus formyl can be removed by peptide deformylase (PDF) [18]. The enzymatic activity of PDF requires iron, but once the iron in the culture medium is depleted, PDF activity will be inhibited, resulting in the failure of fδ-toxin to be deformylated, and the proportion of fδ-toxin in the supernatant will increase [52]. Moreover, in our studies at room temperature, rather than 37°C, the proportion of dfδ-toxin was significantly decreased (data not shown). We noted that this may be a way for *S. aureus* to regulate its physiological state. When the environment is rich in nutrients and suitable for growth, more dfδ-toxin is produced to promote expansion, but when environmental conditions worsen, more fδ-toxin is produced to promote *S. aureus* defense against adverse conditions. The formylation and deformylation of δ-toxin involves a very low-cost yet effective way to regulate the physiological, and even the multi-toxin, state of *S. aureus*.

Importantly, we found that in some disease mice models, reduction of the pathogenic effect of Δδ-toxin was not so substantial, which may be why the function of δ-toxin had been unclear. We speculate that some common mice models often involve a relatively large number of bacteria, and the powerful pathogenic function of many other toxins will obscure the importance of δ-toxin. For example, when δ-toxin cannot be produced, PSMα may not be transported into the supernatant, but it could still perform its original function on the cell surface. Therefore, in some mice models, the Δδ-toxin only showed a delay rather than a decrease in effect, which suggests that the importance of δ-toxin is more likely associated with repeated or chronic infections, rather than acute infections [53]. Verification of this conjecture requires more comprehensive and in-depth study.

In our experiments on synthetic peptides, we found that fδ-toxin tends to form fibrils and dfδ-toxin tends to form oligomers, but the situation in natural environments will be more complicated. When using HPLC to detect fδ-toxin and dfδ-toxin concentrations in aggregates, we found that there is a certain amount of dfδ-toxin (~45%) in the fibrils of Aggregate1, and also a small amount of fδ-toxin (~10%) in the oligomers of Aggregate2 (Figure 5(a-d)). We speculate that the combination of fδ-toxin monomer and dfδ-toxin monomer may fluctuate random. They will form oligomers, but when an oligomer has a few fδ-toxin monomers, it can maintain its state as an oligomer. On the contrary, when the ratio of fδ-toxin monomer becomes larger, it will continue to aggregate, thereby forming fibrils, rather than remaining oligomeric.

PSMα3 is the most cytolytic PSM produced by *S. aureus* [54]. This is why it has been the focus of most investigation on PSMs, including the mechanism by which it causes disease, and structure-function relationship studies [12]. Some results show that PSMs penetrate the cell membrane, most likely by transient pore formation instead of receptor [55,56]. However, the mechanism PSMα uses to interact with membranes is not clear. Of note, although the ability of PSMα3 to form fibers has been generally recognized, there are also differing reports on whether or not PSMα3 fibrils exert cytotoxicity. Some reports showed that PSMα3 fibrils facilitate cytotoxicity [22,57], and provide mechanistic insight into species-specific toxicity of a key bacterial amyloid virulence factor via reciprocal interactions with membranes [58]. However, there are also reports attesting that amyloid formation is not linked to PSM cytotoxicity [59,60]. In the study of amphipathic peptides, such as Aβ_42_, it was found that such oligomers may represent the primary toxic species of amyloids [61]. In our research, we also found a similar oligomer in the purified product of the PSMs, and it is important that this oligomer is a complex mainly composed of dfδ-toxin and PSMα, which showed increased cytotoxicity. Thus, we propose that the oligomer complex formed by dfδ-toxin and PSMα may be an important modification of PSMα allowing it to better perform its functions. Because Aggregate2 of ΔPSMα is an 8 monomer oligomer mainly composed of dfδ-toxin, and it is of similar size as the Aggregate2 of MW2, we speculate that the binding of PSMα and dfδ-toxin will be based on 8-mer dfδ-toxin oligomers. However, some possible combination of PSMα partially replacing dfδ-toxin in 8-mer oligomers, or of PSMα combining with 8-mer oligomers of dfδ-toxin, or also adopting some even more complicated schemes requires more in-depth research.

This study opens new perspectives into PSMα oligomer-related cytotoxicity. But what is puzzling is that in the synthetic peptide Co-IP results, we found that both dfδ-toxin and fδ-toxin can bind to PSMα and enhance cytotoxicity (Figure 10(a-b), Table 4). However, what we found in the purified supernatant was mainly dfδ-toxin bound to PSMα (Figure 4(a), Figure 5(a-d), Table 2). The reasons for this are still unclear. We speculate that the assembly of δ-toxin and PSMα may be a dynamic process. Although PSMα can bind to both δ-toxins, only fδ-toxin without PSMα can successful aggregate oligomers to form fibrils. While dfδ-toxin mainly exists in the oligomeric form, PSMα can be smoothly combined with dfδ-toxin to maintain this state, this dynamic reaches equilibrium in the supernatant. Therefore, the final result is that PSMα is mainly found combined with dfδ-toxin.

In the results, we speculate that the secondary structure of fδ-toxin and dfδ-toxin leads to the difference in oligomers: fδ-toxin oligomers can continue to assemble into fibers, while dfδ-toxin oligomers will remain oligomeric. Therefore, fδ-toxin tends to form fibers, while dfδ-toxin tends to form oligomers. In some studies on amyloid peptides, it also has been reported that terminal capping of an amyloidogenic peptide affects fibrillation propensity and fibril morphology [62]. In addition to the difference in the secondary structure, the terminal modification may create an interaction force with other positions of the polypeptide during the aggregation process. Interaction forces are generated in other locations, which affects the aggregate situation. Research on amyloids related to human neurodegenerative diseases has always attracted considerable attention. In recent years, the amyloid-like inclusions found in prokaryotes have given new inspiration to the study of amyloid-related disease. In the view of some investigators, molecular mimicry between bacterial and human amyloids contributes to the pathology of aggregation diseases by direct amyloid interactions and cross-seeding, and/or microbial amyloid interactions with the immune system [63]. In Alzheimer’s disease, the metastable and polydispersed properties of Aβ_42_ oligomerization makes it study extremely difficult. In our research, we found a relatively stable oligomer that exists naturally, but this stability is broken upon N-terminus formyl modification. Therefore, the dfδ-toxin oligomer and the N-terminus formyl modification are likely to contribute to research on protein aggregation, biomaterial design, and/or the study of oligomerization.

## Data Availability

The authors confirm that the data supporting the findings of this study are available within the article.
